# Young man with severe metabolic acidosis after transformer oil ingestion: a case report

**DOI:** 10.1186/s13256-018-1827-4

**Published:** 2018-10-10

**Authors:** Farah Gul Khan, Syed Muhammad Zubair

**Affiliations:** Department of Medicine, Aga Khan University Hospital, Karachi, Pakistan

**Keywords:** Transformer oil, Acute kidney injury, Metabolic acidosis, Anion gap, Toxicity

## Abstract

**Background:**

Transformer oil is used in oil-filled transformers for its insulating as well as coolant properties. Transformer oil ingestion for attempted suicide is seldom heard of. Our patient’s case presented us with a major diagnostic as well as treatment challenge because we encountered such a case for the first time and were totally unaware of the fact that methanol might make up the main component of an aged transformer oil.

**Case presentation:**

A 19-year-old Pakistani/Asian man was brought to our hospital with altered sensorium. He was found to have elevated anion gap acidosis, increased osmolal gap, and acute kidney injury. He had no evidence of rhabdomyolysis or hemolysis. Computed tomography of his head showed cerebral edema. He was resuscitated with intravenous fluids and bicarbonate. Three days later, he confessed taking transformer oil with suicidal intention. His clinical picture mimicked acute methanol intoxication. With an initial improvement in his neurological status, he started complaining of constant headache with episodes of agitation and delirium. His renal function continued worsening despite an adequate urine output. He showed a remarkable improvement in his neurological state after just one session of hemodialysis.

**Conclusions:**

There is evidence that aged transformer oil contains methanol, and a patient who consumes it can present with features mimicking acute methanol intoxication.

## Background

The field of medicine is very challenging but rewarding at the same time. Taking a history from patients with altered sensorium is a difficult task for physicians, especially if the clinical scenario points to substance abuse. Moreover, if the contents of a substance are not known, diagnosis and treatment are further delayed. A combination of high anion gap metabolic acidosis and elevated osmolal gap often points to acute ethanol, methanol, or ethylene glycol poisoning that can be confirmed only by a history of ingestion of these products or positive blood tests. However, the absence of either of these poses the biggest diagnostic dilemma, because they are only indicative and not pathognomonic of acute methanol/ethylene glycol toxicity.

We report a case of a young man with altered mental status and high osmolal and anion gap metabolic acidosis along with acute kidney injury who later confessed taking transformer oil in a suicide attempt. The transformer oil had been lying in his home for more than 1 year. Transformer oil is used in oil-filled transformers for its insulating as well as coolant properties. Transformer oil ingestion for attempted suicide is seldom heard of. This case presented us with a major diagnostic as well as treatment challenge because we encountered such a case for the first time and were totally unaware of the fact that methanol might make up the main component of an aged transformer oil.

## Case presentation

A 19-year-old Pakistani/Asian man with a low socioeconomic background was brought to the emergency department of our hospital with a 15-h history of altered behavior, acute confusion, and disturbed gait. His family did not report any fever, recent fall, accident, or substance abuse. His parents had died at a young age, and he was living with his paternal uncle. He used to work in a generator shop, and he had a history of occasional alcohol and cannabis intake and benzodiazepine abuse 6 months earlier.

On presentation, his blood pressures was 148/65 mmHg with a regular heart rate 96 beats/min. His oxygen saturation was normal, but his breathing was rapid and deep at a rate of 32/min. His temperature was recorded at 36.8 °C. On examination, he was found to be very agitated and was not comprehending. His neck was supple, and his examination result was negative for Kernig’s and Brudzinski’s signs. He was moving all four limbs symmetrically and withdrawing from painful stimuli. His tendon reflexes were normal bilaterally, and his plantar responses were downward. His pupils were normal in size and equally reactive to light. The results of his chest, abdominal, and cardiac examinations were within normal limits.

Laboratory investigations showed serum anion gap 28 mmol/L, osmolal gap 22.5 mOsmol/kg, arterial pH 7.23, lactate 15 mmol/L, potassium 5.6 mmol/L, sodium 140 mmol/L, bicarbonate 5.8 mmol/L, random blood sugar 108 mg/dl, serum blood urea nitrogen (BUN) 7 mg/dl, serum creatinine 1.3 mg/dl, hemoglobin 17 g/dl, white blood cell count 24.4 × 10^9^ (neutrophils 82%), platelets 447 × 10^9^, negative urine toxicology screen (amphetamine, cannabinoids, barbiturates, benzodiazepines, opiates, and cocaine), and negative serum ethanol. Serum methanol levels were not measured, because the assay was not available. urinalysis demonstrated 2+ proteins, 1 white blood cell, 10 red blood cells, 5+ hemoglobin, no cast, and no crystals. The results of amylase, lipase, creatinine phosphokinase, and liver function tests, including alanine aminotransferase, aspartate aminotransferase, and alkaline phosphatase, were within normal ranges. Blood and urine culture results were negative.

The patient’s chest radiography result was normal. His electrocardiogram showed sinus tachycardia. Ultrasound of his kidneys revealed bilateral swollen kidneys. Computed tomography (CT) of his head showed cerebral edema.

At that point, our differential diagnosis included acute methanol or ethylene glycol poisoning (on the basis of high anion gap metabolic acidosis; elevated osmolal gap; and history of substance abuse in the past, though there was no history of intake) and septic encephalopathy (acute confusional state with raised white blood cell count).

In the presence of severe metabolic acidosis and acute kidney injury, the patient was started on an intravenous diluted sodium bicarbonate infusion along with intravenous crystalloids. Empiric intravenous ceftriaxone was initiated.

During the first 24 hours of admission, the patient showed remarkable improvement in his consciousness level; however, he was still delirious but started following commands. His serum bicarbonate improved to 18 mEq/L, and his white blood cell count decreased. Although his urine output was adequate at approximately 1.2–1.5 L/d, his serum creatinine worsened. The family and the patient were questioned again regarding intake of methanol or ethylene glycol, but denied it completely.

On day 3 of admission, the patient confessed taking transformer oil in order to commit suicide. The transformer oil had been kept in their home for more than 1 year and was collected from a burst transformer. On subsequent days, after an initial improvement, the patient’s consciousness level deteriorated rapidly, and he started complaining of continuous headache with episodes of increased agitation alternating with acute delirium. Despite an adequate urine output and normal electrolytes, his serum creatinine kept on worsening.

At that time, his consciousness level was not explained by uremia, because his BUN was only 50 mEq/L, though his creatinine had risen to 8.5 mg/dl. Hemodialysis was considered at that point, but our suspicion was intake of some lipophilic substance that could not be dialyzed. Because of the patient’s unexplained delirious state, CT of the head was repeated, which revealed abnormal low-density areas in bilateral temporal, parietal, and occipital lobes; genu of corpus callosum; and right cerebellar peduncle along with cerebral edema.

On day 7, the patient’s urine output declined, and his BUN and creatinine increased to 80 mg/dl and 12.5 mg/dl, respectively. He had two episodes of generalized tonic-clonic seizures and was dialyzed immediately for 4 hours. After the first hemodialysis session, although the patient’s BUN fell only slightly from 80 to 60 mg/dl, he showed a remarkable improvement in his consciousness level. He became calm and alert, his agitation was almost alleviated, and his complaints of headache became less frequent. He was again dialyzed consecutively for the next 2 days.

After three consecutive sessions of hemodialysis, no further dialysis was needed. The patient’s headache subsided completely, and his consciousness level improved significantly, which further pointed to removal of certain substances via dialysis as the cause of altered sensorium and kidney injury. He was discharged on day 12 with stable serum creatinine, which was completely normalized 10 days after discharge. The patient is under regular follow-up, and his renal function is normal. The trend of the patient’s renal function is shown in Table 1.

**Table 1:** Laboratory test results

Laboratory test	Day 1	Day 3	Day 5	Day 7		Day 8	Days 9 and 10	Day 12 (discharged to home)	Day 17 (follow-up visit)	Day 23 (follow-up visit)
BUN (mg/dl)	7	37	53	80	Dialyzed	63	Dialyzed two times	37	18	
Creatinine (mg/dl)	1.3	5.7	8.6	12.5		10.2		5.3	1.7	1.1
Anion gap	28.2	10.8	14	17.4		15.6		13.6	14	
Urine DR	Protein 2+ Hb 5+ RBC 10 No cast or crystals		Protein 4+ Hb 4+ RBC > 20 WBC > 20 No cast or crystals							Protein negative Hb negative RBC negative No cast or crystals

*Abbreviations: BUN* Blood urea nitrogen, *Hb* Hemoglobin, *RBC* Red blood cells, *WBC* White blood cells

## Discussion

Our patient reported ingestion of transformer oil in a suicide attempt and presented with altered sensorium, elevated osmolal gap, high anion gap metabolic acidosis, and acute kidney injury. With initial improvement in his consciousness level, he started complaining of continuous headache and became confused and delirious with worsening kidney function. His clinical condition and laboratory parameters mimicked acute methanol intoxication, and he showed phenomenal improvement in his consciousness level after just a single hemodialysis session. Our patient’s case is unique because, to the best of our knowledge, this is the first case in the literature of transformer oil poisoning presenting with high osmolal gap, anion gap acidosis, and acute kidney injury mimicking acute methanol intoxication. Our patient posed a major diagnostic and treatment challenge because we were unaware that methanol is present in aged transformer oil and that the patient’s presentation can be similar to acute methanol intoxication [1].

Transformer oil poisoning is a very rare type of chemical poisoning. Most of the time, it presents with the sign and symptoms of chronic poisoning and affects people employed as electrical pole workers or who live near incinerators or other disposal facilities [2]. In Southeast Asia, people get this oil very easily and sometimes use it for massage in order to relieve pain. Occasionally, it is also used as a cooking oil for food adulteration [3].

Polychlorinated biphenyls (PCBs) were widely used as transformer oil; however, because of their toxicity, carcinogenicity, and bioaccumulativeness, they were banned a long time ago. Some developing countries in Asia and Africa are still using it for industrial and commercial purposes despite a worldwide ban on PCBs [4].

Field transformer oil consists of many components, such as methyl acetate, phenol, 2-methylfuran, methyl formate, furan, ethanol, methanol, isopropyl alcohol, acetone, and methyl ethyl ketone. The last five of these components are alcohols and ketones [5]. In oil-filled power transformers, cellulose-based material is widely used for insulation. The cellulose degradation is used to detect the end of the life of a transformer. This degradation leads to short-circuiting between conductors and ultimately loss of equipment. Direct evaluation of cellulose degradation in a working transformer is impractical; however, indirect evaluation can be done by measuring ethanol and methanol in transformer oil, which serve as chemical markers for aging [6]. The transformer oil that our patient ingested was collected from a burst transformer, and the clinical findings and laboratory investigations mimicked acute methanol poisoning. Serum analysis on admission revealed high anion gap metabolic acidosis along with an increased osmolal gap, consistent with the presence in the blood of a low-molecular-weight substance. Our patient had altered sensorium and acute kidney injury on admission that rapidly deteriorated within a span of few days.

Although the kidneys generally are not considered as a primary target organ in methanol intoxication, a number of studies have shown that, apart from myoglobinuria and hemolysis, the kidneys may be involved owing to a number of factors, such as tissue hypoxia and injury because of inhibition of mitochondrial cytochrome oxidase, as well as vacuolar distention of proximal cells and direct injury of proximal cells because of high serum osmolality. Osmotic injury may cause reversible low-grade proteinuria, as seen in our patient [7–9].

The signs and symptoms of methanol intoxication are nonspecific and inconsistent. Symptoms usually include nausea, vomiting, abdominal pain, headache, impaired sensorium, and impaired vision. However, visual disturbance was not present in our patient. Rastogi *et al.* reported a case of a patient with severe methanol intoxication after airplane fuel ingestion who did not experience any visual impairment and had no optic papillitis [1]. In another study, 37% of patients experienced visual disturbance with methanol intoxication [10]. There is only one case report in the literature from Bangladesh, which describes a young female who presented with severe noncardiogenic pulmonary edema after transformer oil ingestion [11]. Unlike our patient, she did not develop kidney injury and was managed with diuretics.

## Conclusions

Our patient’s case is the first-ever case, to the best of our knowledge, of transformer oil poisoning presenting with severe renal failure. Transformer oil contains methanol, the concentration of which increases with the aging of oil; thus, a patient can present with clinical and laboratory findings mimicking acute methanol intoxication.

## Abbreviations

BUN: Blood urea nitrogen
CT: Computed tomography
Hb: Hemoglobin
PCB: Polychlorinated biphenyl
RBC: Red blood cell(s)
WBC: White blood cell(s)
